# 2-Bromo-4-chloro-6-[(*E*)-*o*-tolyl­imino­meth­yl]phenol

**DOI:** 10.1107/S1600536810037931

**Published:** 2010-09-30

**Authors:** Jin-Bao Guo

**Affiliations:** a Department of Chemistry and Chemical Engineering, Baoji University of Arts and Sciences, Baoji, Shaanxi 721007, People’s Republic of China

## Abstract

The title compound, C_14_H_11_BrClNO, is a Schiff base compound derived from the condensation of 3-bromo-5-chloro­salicyl­aldehyde and *o*-toluidine in methanol. The aromatic rings make a dihedral angle of 38.3 (1)°. The mol­ecular conformation is stabilized by an intra­molecular O—H⋯N hydrogen bond, generating an *S*(6) ring.

## Related literature

For Schiff bases, see: Ali *et al.* (2002 ▶). For related structures, see: Li & Zhang (2005 ▶, 2006 ▶); Li *et al.* (2006 ▶).
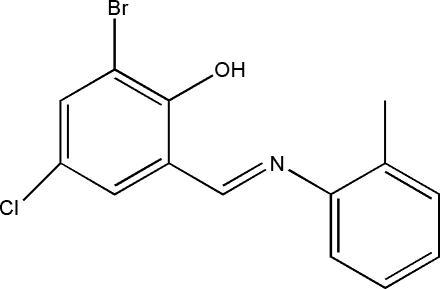


## Experimental

### 

#### Crystal data

C_14_H_11_BrClNO
*M* *_r_* = 324.60
Orthorhombic, 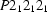

*a* = 7.5388 (9) Å
*b* = 12.2452 (11) Å
*c* = 14.2440 (16) Å
*V* = 1314.9 (2) Å^3^
*Z* = 4
Mo *K*α radiation
μ = 3.32 mm^−1^
*T* = 298 K
0.40 × 0.38 × 0.33 mm

#### Data collection

Bruker SMART CCD area-detector diffractometer
Absorption correction: multi-scan (*SADABS*; Bruker, 2003 ▶) *T*_min_ = 0.351, *T*_max_ = 0.408
5422 measured reflections
2284 independent reflections
1815 reflections with *I* > 2σ(*I*)
*R*_int_ = 0.031

#### Refinement

*R*[*F* ^2^ > 2σ(*F* ^2^)] = 0.032
*wR*(*F* ^2^) = 0.057
*S* = 1.09
2284 reflections
164 parameters
H-atom parameters constrained
Δρ_max_ = 0.25 e Å^−3^
Δρ_min_ = −0.33 e Å^−3^
Absolute structure: Flack (1983 ▶), 938 Friedel pairs
Flack parameter: 0.006 (10)

### 

Data collection: *SMART* (Bruker, 2003 ▶); cell refinement: *SAINT* (Bruker, 2003 ▶); data reduction: *SAINT*; program(s) used to solve structure: *SHELXS97* (Sheldrick, 2008 ▶); program(s) used to refine structure: *SHELXL97* (Sheldrick, 2008 ▶); molecular graphics: *SHELXTL* (Sheldrick, 2008 ▶); software used to prepare material for publication: *SHELXTL*.

## Supplementary Material

Crystal structure: contains datablocks I, global. DOI: 10.1107/S1600536810037931/bt5360sup1.cif

Structure factors: contains datablocks I. DOI: 10.1107/S1600536810037931/bt5360Isup2.hkl

Additional supplementary materials:  crystallographic information; 3D view; checkCIF report

## Figures and Tables

**Table 1:** Hydrogen-bond geometry (Å, °)

*D*—H⋯*A*	*D*—H	H⋯*A*	*D*⋯*A*	*D*—H⋯*A*
O1—H1⋯N1	0.82	1.86	2.588 (3)	147

**Fig. 1.**
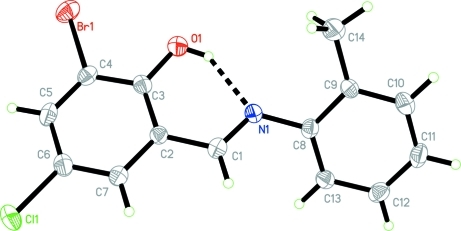
The molecular structure of the title compound, showing 50% probability displacement ellipsoids. The dashed line indicates a hydrogen bond.

**Fig. 2.**
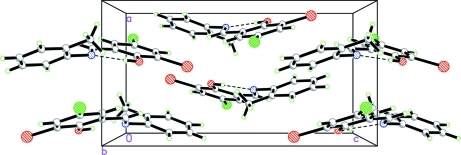
The crystal packing of the title compound, viewed along the b axis.

## supplementary crystallographic information

### Comment

The roles of Schiff base compounds in biological processes have become a topic of study in recent years. Schiff base compounds have demonstrated significant biological activity and new examples are being tested for their antitumor, antimicrobial, and antiviral activity (Ali *et al.*, 2002). In the past years, we have prepared a series of Schiff base cmpounds, and investigated their structure and properties (Li *et al.*, 2006; Li & Zhang, 2006; Li & Zhang, 2005).

In the title compound (Fig. 1), all the bond lengths and angles are within normal value   and are comparable to those observed in a similar Schiff base compound (Li & Zhang, 2005). The two aromatic rings are linked by a C ═N bond and enclose a dihedral angle of 38.3 (1) °. As expected, the molecule adopts a *trans* configuration about the C1 ═N1 bond. The molecular conformation is stabilized by an intramolecular O—H–N hydrogen bond (Table 1).

### Experimental

3-bromo-5-Chlorosalicylaldehyde (0.1 mmol, 23.5 mg) and *o*-toluidine (0.1 mmol, 10.7 mg) dissolved in MeOH (10 ml). The solution was stirred for half an hour and then filtered. Crystals of the title compound suitable for single-crystal X-ray analysis were recrystallized from methanol after one weeks at room temperature. The yellow block precipitate was filtered, washed with cold MeOH, and dried *in vacuo* for 48 h (yield 70%).

### Crystal data

**Table:**

C_14_H_11_BrClNO	*D*_x_ = 1.640 Mg m^−^^3^
*M**_r_* = 324.60	Mo *K*α radiation, λ = 0.71073 Å
Orthorhombic, *P*2_1_2_1_2_1_	Cell parameters from 2013 reflections
*a* = 7.5388 (9) Å	θ = 2.2–23.7°
*b* = 12.2452 (11) Å	µ = 3.32 mm^−^^1^
*c* = 14.2440 (16) Å	*T* = 298 K
*V* = 1314.9 (2) Å^3^	Block, yellow
*Z* = 4	0.40 × 0.38 × 0.33 mm
*F*(000) = 648	

### Data collection

**Table:**

Bruker SMART CCD area-detector diffractometer	2284 independent reflections
Radiation source: fine-focus sealed tube	1815 reflections with *I* > 2σ(*I*)
graphite	*R*_int_ = 0.031
φ and ω scans	θ_max_ = 25.0°, θ_min_ = 2.2°
Absorption correction: multi-scan (*SADABS*; Bruker, 2003)	*h* = −8→8
*T*_min_ = 0.351, *T*_max_ = 0.408	*k* = −14→14
5422 measured reflections	*l* = −16→8

### Refinement

**Table:**

Refinement on *F*^2^	Secondary atom site location: difference Fourier map
Least-squares matrix: full	Hydrogen site location: inferred from neighbouring sites
*R*[*F*^2^ > 2σ(*F*^2^)] = 0.032	H-atom parameters constrained
*wR*(*F*^2^) = 0.057	*w* = 1/[σ^2^(*F*_o_^2^) + (0.0039*P*)^2^] where *P* = (*F*_o_^2^ + 2*F*_c_^2^)/3
*S* = 1.09	(Δ/σ)_max_ = 0.001
2284 reflections	Δρ_max_ = 0.25 e Å^−^^3^
164 parameters	Δρ_min_ = −0.33 e Å^−^^3^
0 restraints	Absolute structure: Flack (1983), 938 Friedel pairs
Primary atom site location: structure-invariant direct methods	Flack parameter: 0.006 (10)

### Special details

**Table:**

Geometry. All e.s.d.'s (except the e.s.d. in the dihedral angle between two l.s. planes) are estimated using the full covariance matrix. The cell e.s.d.'s are taken into account individually in the estimation of e.s.d.'s in distances, angles and torsion angles; correlations between e.s.d.'s in cell parameters are only used when they are defined by crystal symmetry. An approximate (isotropic) treatment of cell e.s.d.'s is used for estimating e.s.d.'s involving l.s. planes.
Refinement. Refinement of *F*^2^ against ALL reflections. The weighted *R*-factor *wR* and goodness of fit *S* are based on *F*^2^, conventional *R*-factors *R* are based on *F*, with *F* set to zero for negative *F*^2^. The threshold expression of *F*^2^ > σ(*F*^2^) is used only for calculating *R*-factors(gt) *etc*. and is not relevant to the choice of reflections for refinement. *R*-factors based on *F*^2^ are statistically about twice as large as those based on *F*, and *R*- factors based on ALL data will be even larger.

### Fractional atomic coordinates and isotropic or equivalent isotropic displacement parameters (Å^2^)

**Table:**

	*x*	*y*	*z*	*U*_iso_*/*U*_eq_	
Br1	0.04915 (6)	0.84654 (3)	−0.29615 (2)	0.06176 (15)	
Cl1	0.26999 (17)	1.17472 (7)	−0.05785 (7)	0.0692 (3)	
N1	0.1302 (3)	0.6835 (2)	0.05868 (17)	0.0362 (7)	
O1	0.0835 (3)	0.71558 (18)	−0.11917 (15)	0.0500 (7)	
H1	0.0929	0.6804	−0.0704	0.075*	
C1	0.1777 (5)	0.7832 (3)	0.0667 (2)	0.0388 (9)	
H1A	0.2136	0.8093	0.1250	0.047*	
C2	0.1770 (4)	0.8568 (3)	−0.0137 (2)	0.0339 (8)	
C3	0.1263 (4)	0.8203 (3)	−0.1027 (2)	0.0376 (9)	
C4	0.1196 (5)	0.8952 (3)	−0.1764 (2)	0.0400 (9)	
C5	0.1624 (5)	1.0029 (3)	−0.1627 (2)	0.0450 (10)	
H5	0.1554	1.0522	−0.2122	0.054*	
C6	0.2160 (5)	1.0382 (3)	−0.0746 (2)	0.0447 (10)	
C7	0.2229 (5)	0.9665 (3)	−0.0010 (2)	0.0408 (9)	
H7	0.2583	0.9909	0.0579	0.049*	
C8	0.1220 (5)	0.6153 (3)	0.1403 (2)	0.0364 (9)	
C9	0.1705 (5)	0.5059 (3)	0.1305 (2)	0.0360 (9)	
C10	0.1639 (5)	0.4404 (3)	0.2096 (3)	0.0477 (9)	
H10	0.1993	0.3678	0.2053	0.057*	
C11	0.1059 (5)	0.4805 (3)	0.2947 (3)	0.0516 (11)	
H11	0.1014	0.4348	0.3467	0.062*	
C12	0.0553 (5)	0.5866 (3)	0.3027 (2)	0.0478 (9)	
H12	0.0163	0.6133	0.3602	0.057*	
C13	0.0618 (5)	0.6549 (3)	0.22552 (19)	0.0415 (8)	
H13	0.0259	0.7273	0.2309	0.050*	
C14	0.2313 (6)	0.4607 (3)	0.0376 (2)	0.0600 (12)	
H14A	0.2589	0.3845	0.0445	0.090*	
H14B	0.3352	0.4993	0.0171	0.090*	
H14C	0.1387	0.4692	−0.0081	0.090*	

### Atomic displacement parameters (Å^2^)

**Table:**

	*U*^11^	*U*^22^	*U*^33^	*U*^12^	*U*^13^	*U*^23^
Br1	0.0701 (3)	0.0765 (3)	0.03869 (18)	0.0032 (3)	−0.0065 (2)	0.0004 (2)
Cl1	0.1052 (9)	0.0331 (5)	0.0692 (6)	−0.0064 (6)	−0.0041 (6)	0.0075 (5)
N1	0.0390 (18)	0.0323 (16)	0.0372 (15)	−0.0012 (14)	0.0006 (13)	0.0021 (14)
O1	0.064 (2)	0.0434 (14)	0.0423 (13)	−0.0024 (13)	−0.0035 (13)	−0.0011 (11)
C1	0.034 (2)	0.045 (2)	0.038 (2)	0.0001 (18)	0.0003 (18)	0.0037 (18)
C2	0.035 (2)	0.033 (2)	0.0335 (17)	0.0014 (19)	0.0021 (15)	0.0014 (18)
C3	0.033 (2)	0.034 (2)	0.046 (2)	0.0048 (17)	0.0093 (16)	−0.0010 (18)
C4	0.036 (2)	0.050 (2)	0.034 (2)	0.0074 (18)	0.0019 (16)	0.0027 (17)
C5	0.045 (3)	0.050 (2)	0.040 (2)	0.009 (2)	0.0088 (19)	0.0144 (19)
C6	0.045 (3)	0.038 (2)	0.050 (2)	0.001 (2)	0.0066 (19)	0.006 (2)
C7	0.045 (3)	0.041 (2)	0.0354 (19)	−0.002 (2)	0.0011 (17)	0.0011 (18)
C8	0.038 (2)	0.036 (2)	0.0354 (19)	−0.0042 (16)	−0.0018 (16)	0.0049 (16)
C9	0.039 (2)	0.030 (2)	0.0396 (19)	−0.0054 (18)	−0.0004 (18)	0.0001 (17)
C10	0.052 (3)	0.033 (2)	0.058 (2)	−0.0044 (18)	−0.001 (2)	0.004 (2)
C11	0.063 (3)	0.051 (3)	0.041 (2)	−0.010 (2)	−0.006 (2)	0.017 (2)
C12	0.054 (3)	0.053 (2)	0.0363 (19)	−0.012 (2)	0.005 (2)	0.0002 (19)
C13	0.049 (2)	0.0355 (19)	0.0402 (18)	0.002 (2)	0.0055 (17)	−0.0007 (17)
C14	0.075 (3)	0.050 (2)	0.055 (2)	0.000 (3)	0.015 (2)	0.000 (2)

### Geometric parameters (Å, °)

**Table:**

Br1—C4	1.883 (3)	C7—H7	0.9300
Cl1—C6	1.737 (4)	C8—C13	1.383 (4)
N1—C1	1.277 (4)	C8—C9	1.395 (4)
N1—C8	1.433 (4)	C9—C10	1.385 (4)
O1—C3	1.343 (4)	C9—C14	1.506 (5)
O1—H1	0.8200	C10—C11	1.378 (5)
C1—C2	1.458 (4)	C10—H10	0.9300
C1—H1A	0.9300	C11—C12	1.359 (5)
C2—C7	1.398 (5)	C11—H11	0.9300
C2—C3	1.398 (4)	C12—C13	1.382 (4)
C3—C4	1.394 (4)	C12—H12	0.9300
C4—C5	1.373 (5)	C13—H13	0.9300
C5—C6	1.386 (4)	C14—H14A	0.9600
C5—H5	0.9300	C14—H14B	0.9600
C6—C7	1.368 (4)	C14—H14C	0.9600
			
C1—N1—C8	119.8 (3)	C13—C8—N1	121.5 (3)
C3—O1—H1	109.5	C9—C8—N1	117.8 (3)
N1—C1—C2	121.3 (3)	C10—C9—C8	117.7 (3)
N1—C1—H1A	119.3	C10—C9—C14	120.8 (3)
C2—C1—H1A	119.3	C8—C9—C14	121.4 (3)
C7—C2—C3	119.4 (3)	C11—C10—C9	121.3 (3)
C7—C2—C1	119.5 (3)	C11—C10—H10	119.3
C3—C2—C1	121.1 (3)	C9—C10—H10	119.3
O1—C3—C4	119.2 (3)	C12—C11—C10	120.2 (3)
O1—C3—C2	121.9 (3)	C12—C11—H11	119.9
C4—C3—C2	118.9 (3)	C10—C11—H11	119.9
C5—C4—C3	121.0 (3)	C11—C12—C13	120.1 (3)
C5—C4—Br1	120.0 (3)	C11—C12—H12	119.9
C3—C4—Br1	119.0 (3)	C13—C12—H12	119.9
C4—C5—C6	119.8 (3)	C12—C13—C8	119.8 (3)
C4—C5—H5	120.1	C12—C13—H13	120.1
C6—C5—H5	120.1	C8—C13—H13	120.1
C7—C6—C5	120.3 (3)	C9—C14—H14A	109.5
C7—C6—Cl1	120.2 (3)	C9—C14—H14B	109.5
C5—C6—Cl1	119.5 (3)	H14A—C14—H14B	109.5
C6—C7—C2	120.5 (3)	C9—C14—H14C	109.5
C6—C7—H7	119.7	H14A—C14—H14C	109.5
C2—C7—H7	119.7	H14B—C14—H14C	109.5
C13—C8—C9	120.7 (3)		
			
C8—N1—C1—C2	−176.1 (3)	Cl1—C6—C7—C2	179.0 (3)
N1—C1—C2—C7	177.3 (3)	C3—C2—C7—C6	0.9 (5)
N1—C1—C2—C3	−0.9 (5)	C1—C2—C7—C6	−177.3 (3)
C7—C2—C3—O1	179.0 (3)	C1—N1—C8—C13	38.4 (5)
C1—C2—C3—O1	−2.8 (5)	C1—N1—C8—C9	−144.0 (3)
C7—C2—C3—C4	−1.3 (5)	C13—C8—C9—C10	−2.8 (5)
C1—C2—C3—C4	176.9 (3)	N1—C8—C9—C10	179.5 (3)
O1—C3—C4—C5	−180.0 (3)	C13—C8—C9—C14	178.3 (3)
C2—C3—C4—C5	0.3 (5)	N1—C8—C9—C14	0.6 (5)
O1—C3—C4—Br1	0.4 (4)	C8—C9—C10—C11	2.1 (5)
C2—C3—C4—Br1	−179.4 (3)	C14—C9—C10—C11	−179.0 (3)
C3—C4—C5—C6	1.1 (5)	C9—C10—C11—C12	−0.7 (6)
Br1—C4—C5—C6	−179.3 (3)	C10—C11—C12—C13	0.0 (6)
C4—C5—C6—C7	−1.4 (6)	C11—C12—C13—C8	−0.8 (6)
C4—C5—C6—Cl1	180.0 (3)	C9—C8—C13—C12	2.2 (6)
C5—C6—C7—C2	0.4 (5)	N1—C8—C13—C12	179.8 (3)

### Hydrogen-bond geometry (Å, °)

**Table:**

*D*—H···*A*	*D*—H	H···*A*	*D*···*A*	*D*—H···*A*
O1—H1···N1	0.82	1.86	2.588 (3)	147
